# Influenza Vaccination Uptake and Associated Factors among Individuals with Diabetes Mellitus in Spain: A Cross-Sectional Study Using Data from the European Health Interview Survey 2020

**DOI:** 10.3390/vaccines12080915

**Published:** 2024-08-13

**Authors:** Eric P. Twomey, David Herman, José A. Marín-Rodríguez, Jose J. Jimenez-Moleon

**Affiliations:** 1 Escuela Andaluza de Salud Pública, Cta. del Observatorio, 4, Beiro, 18011 Granada, Spain; twomey@correo.ugr.es; 2 École des Hautes Études en Santé Publique (EHESP), 20 Av. George Sand, 93210 Paris, France; david.herman@edu.ehesp.fr; 3 Medicina Preventiva y Salud Pública en el Hospital Universitario Virgen de las Nieves, Av. de las Fuerzas Armadas, 2, Beiro, 18014 Granada, Spain; 4 Departamento de Medicina Preventiva y Salud Pública, Faculty of Medicine, Universidad de Granada, Avenida de la Investigación, 11, 18016 Granada, Spain; jjmoleon@ugr.es; 5 Consorcio Centro de Investigación Biomédica en Red de Epidemiología y Salud Pública (CIBERESP), 28029 Madrid, Spain; 6 Instituto de Investigación Biosanitaria ibs.GRANADA, 18012 Granada, Spain

**Keywords:** influenza vaccination, diabetes mellitus, vaccination adherence, EHIS 2020, Spain

## Abstract

Background: Vaccination against influenza has proven to reduce influenza-caused hospital entries, treatment times in intensive care units and hospitalisation costs for treating people with Diabetes Mellitus (DM). Despite the existing influenza vaccination recommendations for all persons with DM, in Spain, vaccination hesitancy remains substantial, and vaccination rates lag behind target. We aimed to assess predictors for influenza vaccination uptake and reasons for non-adherence among individuals with DM. Methods: Data from the 2020 European Health Interview Survey were analysed using uni- and multivariable logistic regression models, stratified by age group and including possible confounders and vaccination as an outcome. Associations with the sociodemographic profile, healthcare access and substance use were explored. Results: Our analysis included 2194 individuals with DM over the age of 15, showing an influenza vaccination rate of 53%. The findings revealed significant predictors of vaccination uptake, including age over 60 years and robust social support. Conversely, younger age, higher education levels, infrequent healthcare interactions and economic barriers emerged as significant obstacles to vaccination. Conclusions: To enhance vaccination rates, targeted public health interventions should emphasise the importance of vaccination for younger, more educated individuals with DM, those facing economic barriers and those with lower levels of social support, which could bridge the existing gap in vaccination coverage.

## 1. Introduction

Influenza vaccination is a crucial health intervention for individuals with Diabetes Mellitus (DM), who are more susceptible to influenza and its complications [1]. This susceptibility is further compounded by the immunocompromised state often associated with DM, highlighting the importance of seasonal influenza vaccination (SIV) which has shown valid protection against influenza in young and old persons with DM (pwDMs), reducing the risk of hospitalisation and mortality [2,3,4]. As recommended by the WHO [5], in Spain, pwDMs are classified as high-risk patients for influenza, leading to targeted public health initiatives in Spain to enhance their protection that specifically include pwDMs in their SIV campaigns, offering free immunisation [6]. At the same time, the Spanish government considers the over-60 age group to be at high risk, and they are therefore recommended to be vaccinated, regardless of additional chronic conditions [6].

Propelled by guidelines issued through health authorities globally [1,5] and within the EU [7,8] and the Spanish government [6], the uptake rates of SIV among pwDMs in Spain increased from 61.4% in 2003 to 63.8% in 2006 and plateaued at 65.0% in 2010 [9]. Recent studies, however, show that the vaccine uptake during 2011 and 2020 among pwDMs in Spain was around 53%, with no significant variation between the years [10], missing the minimum coverage of SIV among pwDMs of ≥75% recommended by the ECDC [8], highlighting a significant necessity for public health action [11]. To address these target levels, possible reasons for vaccine hesitancy from pwDMs towards SIV need to be assessed thoroughly [12].

The influence of sociodemographic factors, such as age and income, and behavioural factors, including healthcare engagement and education, on vaccination uptake has been documented in various studies [13,14,15]. Moreover, the lack of a regular healthcare professional (HCP) and the absence of a professional recommendation have been identified as barriers to SIV [4,16]. Recent publications from South Korea and Hungary investigating factors influencing the uptake of SIV found consistently higher uptakes among older pwDMs [17,18]. Sex and education, however, were only shown to affect SIV uptake in Hungary. In France, age and frequent contact with the healthcare system showed an improvement in SIV uptake rates over time [19].

We aim to examine the factors associated with suboptimal influenza vaccination rates among individuals with DM above and below the age of 60 and identify key determinants that can be utilised in public health strategies to improve coverage using data from the European Health Interview Survey (EHIS), all while adhering to the rigorous standards set forth by the STROBE criteria [20,21].

## 2. Materials and Methods

### 2.1. Study Design and Data Source

A secondary data analysis with data from the cross-sectional EHIS 2020 was conducted. A detailed protocol was published earlier elsewhere [22]. The Eurostat protocol was complemented by the Spanish National Institute for Statistics (INE) whose detailed protocol as well as the corrections for no response due to the COVID-19 pandemic were published elsewhere [23].

Representative population samples were drawn from sampling frames set up at the national level that allowed for persons or households to be selected at random, with a known probability of selection. Spanish data were acquired by a population register with a stratified multi-stage sampling through INE, using a three-step approach based on data from the last census with stratified three-stage sampling, with a proportional random selection of primary and secondary sampling units (towns and sections, respectively), with the final units (non-institutionalised individuals) being selected by means of random routes and sex- and age-based quotas. Eligible participants were individuals at the age of 15 years or above. In Spain, the EHIS was conducted in the time period from July 2019 to July 2020 as a stand-alone study under the name of “Health Interview Study”. The study questionnaire was available in Spanish, Catalan, Valenciano, Euskera, Gallego and English. Data collection was realised by face-to-face interviews and telephone interviews.

### 2.2. Study Population and Variables of Interest

The study population included adults and minors over 15 years old with a diagnosis of DM (see Supplementary Material—Figure S1). The status of DM was fulfilled when participants answered yes to one or more of the following questions: “having a diagnosis of DM”, “having had a former diagnosis of DM” or “medication for diabetes”.

Our outcome of interest was the self-reported non-vaccination during the last SIV, with a binary outcome of ‘Yes’ or ‘No’. In Spain, there is an active recommendation for individuals aged 60 and above to receive the SIV, regardless of the presence of chronic diseases that might further justify vaccination. To account for this potential confounder, we stratified the data by age, dividing the population into those under 60 years and those 60 years and older [24].

Independent covariables encompassed sociodemographic variables, healthcare use, healthcare barriers and psychological and behavioural characteristics that were a priori selected and included in the descriptive analysis and considered for the logistic regression model. Sociodemographic variables included sex [men–women], Spanish nationality [Yes–No], marital status [single, married, widowed, divorced], cohabitation [Yes–No], study level [incomplete primary education, complete primary education, graduate, postgraduate] and social class [directors and managers, intermediate occupations and self-employed, skilled and semi-skilled workers in the primary sector, supervisors and technical skilled workers, unskilled workers]. Healthcare use variables encompassed health perception [good, poor], health insurance [public, private], time of last medical visit [in the last 4 weeks, more than 4 weeks], nurse or midwife consultation [Yes–No], cold medications [Yes–No], use of emergency services [Yes–No] and hospitalisation [Yes–No] and alternative medicine visits (homeopath, acupuncturist, naturist or another alternative medicine) [Yes–No]. Healthcare barriers included no medical attention for economic barriers [Yes–No], no medical attention due to transport barriers [Yes–No] and no medical attention due to Waiting List in the last 12 months [Yes–No]. Psychological and weight status included depressive severity [none, moderate, severe] and body mass index (BMI) [Underweight, Normal, Overweight, Obesity]. Behavioural factors were Physical activity [Sedentary lifestyle, Occasional activity, Monthly activity, Weekly training], alcohol consumption [never or not in the last 12 months, once per month or less, more than once per week], tobacco use [Yes–No] and social support [a lot, somewhat, little or nothing]. Several original categories in depressive severity, social class, study level, health insurance, health perception, alternative medicine visits, marital status, social support, tobacco and alcohol consumption, cohabitation and no medical attention due to transport barriers were grouped together due to the low number of observations.

### 2.3. Statistical Analysis

For the descriptive analyses, each of the categorical variables were presented as the total number and percentage according to the vaccination status. The bivariable association analyses between variables were assessed using chi-square or Fisher’s exact tests as appropriate. Selected participants with no information about any of the covariates were excluded in the bivariable analyses. An additional bivariate analysis was developed for other age groups of interest for vaccination uptake (see Supplementary Material—Table S4).

Using variables with *p* ≤ 0.2 in the bivariate analysis, a stratified multivariable binary logistic regression analysis was performed to identify independent predictors of vaccination in a backward stepwise fashion, by removing not significant variables (*p* > 0.05) except sex and study level as proxy covariates for socioeconomical status, which were mentioned as important confounders for influenza vaccination in the past [14,17,25]. Only significant variables were retained in the final model, with reported non-adjusted (see Supplementary Material—Table S1) and adjusted ORs and associated 95% CI. The log-likelihood, Akaike information criterion and Bayesian information criterion were used to compare the fit of different models.

Similarly to the univariable analyses, the presented multivariable analysis corresponds to a complete case analysis, in which 3.69% (81/2194) of observations with missing values were excluded. No patterns of missing data were detected for each of the covariates both in univariable and multivariable analyses. Nevertheless, substitution analyses with the MICE package were performed for the multivariable model, showing similar results. Collinearity was evaluated using variance inflation factors with a threshold of >5 to indicate significant collinearity (see Supplementary Material—Tables S2 and S3).

Descriptive and logistic regression analyses were performed with R Version 2023.12. R-Code can be accessed online at Git-Hub at https://github.com/davidherman94/vaccination_uptake, last accessed on 4 August 2024.

## 3. Results

To better understand the factors associated with non-vaccination, we analysed the sociodemographic profile, healthcare access, comorbidities and substance use among pwDMs. As pwDMs in Spain above the age of 60 not only have a life-long SIV indication for their DM status but also at least one additional medical indication for SIV (age > 60), to address these potential confounders, we stratified our analysis by these two age groups.

### 3.1. Participants’ Characteristics and Immunisation Status

**Figure 1:**
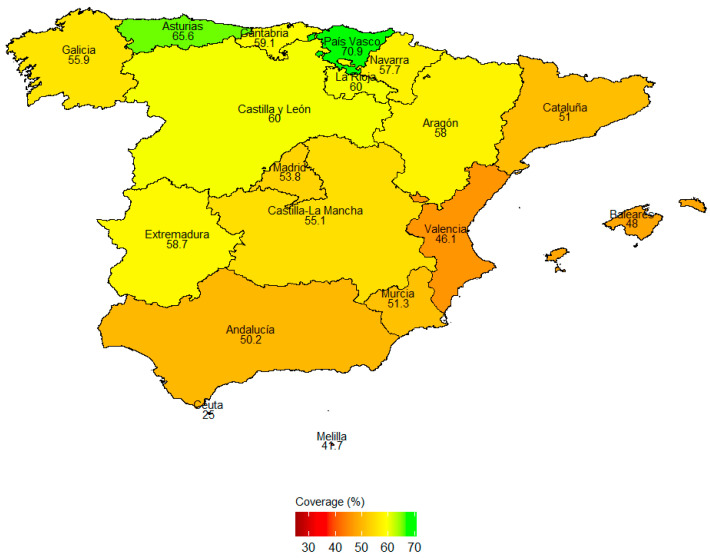
SIV uptake proportions amongst pwDMs in Spain based on the EHIS 2020 data. The provided map offers a visual comparison of influenza vaccination coverage among pwDMs across Spain’s autonomous communities. Substantial regional variations in vaccination uptake, with Ceuta having the lowest (25%) and País Vasco the highest (70.9%) vaccination rates, can be observed. The map serves to identify areas where vaccination rates amongst pwDMs are subpar and targeted efforts and interventions are needed to improve vaccination rates as of 2020. The Canary Islands are not displayed on the map, with a total uptake proportion of 32.9%.

Out of 22,072 participants from the Spanish EHIS 2020 out of the general population >15 years, 5532 (25%) received the SIV. A total of 2193 participants fulfilled inclusion criteria to be considered a pwDM, with 1163 (53%) vaccinated in the last SIV season (Supplementary Figure S1). The mean age among pwDMs was 70.1 years CI 95% (69.6–70.7), with a minimum of 15 and maximum of 95 years. Only one participant was below the Spanish age of medical legal age (16 years). The geographical analysis reveals notable regional disparities, demonstrating a clear north–south gradient (Figure 1). The vaccination coverage ranges significantly across autonomous communities. País Vasco exhibits the highest vaccination rate at 70.9%, while Ceuta has the lowest at 25%. Northern regions such as Asturias (65.6%) and Galicia (59.9%) show higher vaccination rates compared to southern areas like Andalusia (50.2%) and Melilla (47.7%).

### 3.2. Sociodemographic Profile

Age is significantly associated with vaccination uptake, showing 1767 participants over 60 years, where 721 (41%) were unvaccinated, sharply contrasted by the 309 (73%) under 60 years, who remained unvaccinated (*p* < 0.001) (Table 1). Vaccination coverage increases with age, although it is true that coverage is always less than 30% in the population under 60 years of age.

The gender split was almost even, with 1109 (50.5%) men and 1085 (49.5%) women (Table 1). Amongst men, 545 (49%) were unvaccinated; amongst women, 486 (45%) were unvaccinated. However, in the age-stratified model, the results showed that for women under 60 years, the adjusted OR (aOR) for non-vaccination was 0.74 with a 95% CI of 0.46 to 1.18 and a *p*-value of 0.2, indicating no significant difference in the likelihood of vaccination compared to men in this age group (Table 2). For those equal to or over 60 years, the aOR was 1.02 with a 95% CI of 0.83 to 1.25 and a *p* = 0.9, also showing no significant difference in vaccination rates between women and men in this older age group.

Spanish nationality/citizenship suggested differences between Spanish and non-Spanish participants, (46% vs. 74% unvaccinated) (Table 1). However, these differences were not statistically different, nor were they in the model.

Marital status showed that married pwDMs above 60 years of age were significantly (*p* < 0.011) less often unvaccinated (40%) than those that are single (48%) or divorced (52%) (Table 1); nonetheless, there was no statistically significant association shown in the model.

There were 1110 pwDMs with complete primary education, with 502 (45%) being unvaccinated. Graduates numbered 259, with 151 (58%) being unvaccinated, and postgraduates were 283, with 167 (59%) being unvaccinated, interestingly indicating a significant inverse influence of educational attainment on vaccination rates, at least amongst participants older than 60 (*p* < 0.001) (Table 1). This observation was confirmed within the age-stratified model showing that among individuals 60 years or older, compared to those with incomplete primary education, graduates showed again an increased likelihood of non-vaccination (aOR 1.78, 95% CI: 1.24, 2.57, *p* = 0.002), while the effect for complete primary education and postgraduates was not significant (aOR 0.96, 0.76-1.22, *p* = 0.8 and aOR 1.40, 0.98-2.02, *p* = 0.066, respectively). In contrast, in individuals under 60 years, compared to incomplete primary education, those with complete primary education had an aOR of 3.53 (95% CI: 1.49, 8.38, *p* = 0.004), graduates had an aOR of 3.33 (95% CI: 1.31, 8.61, *p* = 0.012) and postgraduates had an aOR of 3.77 (95% CI: 1.45, 9.38, *p* = 0.005) of non-vaccination, indicating that pwDMs with higher education had approximately a three times higher likelihood of not being vaccinated and thus showing even more pronounced results compared to those over 60 years of age (Table 2).

In terms of social support, the analysis showed that 1896 participants reported having a lot of support, with 863 (46%) being unvaccinated. Those reporting somewhat numbered 206, with 113 (55%) being unvaccinated. A smaller group felt little or no support, totalling 79, with 50 (63%) being unvaccinated, indicating the important role of social support in vaccination decisions (Table 1). In the model, this observation could be confirmed within the stratum of people ≥ 60 years, showing that compared to pwDMs with a lot of social support, those with somewhat were 46% (aOR 1.48 CI 95% 1.06–2.07, *p* = 0.023) and those with little or no social support were even 82% (aOR 1.84, CI 95% 1.06–3.18, *p* = 0.034) more likely to not be vaccinated (Table 2).

### 3.3. Healthcare Access

For those with diabetes aged 60 and over, the timing of the last medical visit showed significant variation in non-vaccination rates with pwDMs who visited a medical professional in the last 4 weeks (*n* = 856) showing the lowest frequency of non-vaccination (40%) and those whose visits were longer ago than 4 weeks (*n* = 1336) showing the highest frequency (51%) (Table 1). In the model, individuals 60 years or older, when compared to those who had a visit within the last 4 weeks, the aORs for non-vaccination were 2.31 (95% CI: 1.59, 3.38, *p* < 0.001) for those who had visits 4 weeks or longer ago. However, for pwDMs under 60 years, compared to those who had a visit within the last 4 weeks, those who had a visit 4 weeks or longer ago had an OR of non-vaccination of 1.03 (95% CI: 0.44, 2.40, *p* > 0.9) (Table 2).

Descriptively, pwDMs with past nurse or midwife consultations (*n* = 586) showed significantly lower non-vaccination rates (46%) than those without (*n* = 986) (77%) (Table 1). In the model, not consulting a nurse or midwife was associated with higher non-vaccination rates in the older cohort (aOR 2.22, 95% CI: 1.56, 3.19, *p* < 0.001), compared to the younger group where it was not significant (OR 0.77, 95% CI: 0.36, 1.60, *p* = 0.5) (Table 2).

The previous use of cold medications (*n* = 237) was described to be significantly associated with lower non-vaccination rates (41% vs. 72%) (Table 1). In the model, although not statistically significant, there was a trend suggesting those who did not take cold medications were more likely to not be vaccinated, especially in the older group (OR 1.33, 95% CI: 0.95, 1.88, *p* = 0.1) compared to the younger cohort (OR 1.69, 95% CI: 0.86, 3.26, *p* = 0.12) (Table 2).

Neither health insurance (public 47% vs. private 59%) nor the use of emergency services (45% use vs. 52% no use) showed a statistically significant difference in non-vaccination rates (Table 1).

Hospitalisation within the last 12 months showed a slight and statistically significant difference in non-vaccination rates (42% hospitalised vs. 48% in non-hospitalised) (Table 1), but no association was found in the model.

Concerning economic barriers to medical attention, 2138 participants reported no barriers, with 994 (47%) being unvaccinated. Those facing barriers numbered 55, with 36 (65%) being unvaccinated, indicating economic factors’ impact on vaccination (*p* = 0.007) (Table 1). In the model, this could be confirmed in pwDMs aged 60 years or older showing a higher likelihood of non-vaccination with an aOR of 2.66 (95% CI: 1.34, 5.56, *p* = 0.007), whereas the younger group did not show a significant difference (aOR 1.19, 95% CI: 0.42, 3.76, *p* = 0.8), indicating a higher importance of economic barriers among older pwDMs (Table 2).

### 3.4. Comorbidities and Substance Use

Depressive severity was also considered but showed no significant difference in non-vaccination rates between categories (48% in no depression vs. 44% in moderate depression vs. 58% in severe depression).

Frequent alcohol consumption showed substantially lower vaccination rates (48%) compared to moderate (50%) and no alcohol consumption (57%). Similarly, smokers (49%) showed lower vaccination rates than non-smokers (57%) (Table 1). However, neither alcohol nor tobacco consumption showed statistically significant associations in the model (Table 2).

## 4. Discussion

This study provides a comprehensive analysis of factors associated with non-vaccination against influenza among pwDMs in Spain, employing data from the EHIS 2020. The findings highlight several significant factors associated with vaccination uptake in this specific population.

The descriptive analysis revealed a proportion of SIV uptake within the last season amongst pwDMs in Spain of 53%, while 47% remained unvaccinated. This number deviates strongly from the latest reports on the vaccination uptake of 65%, issued by Jiménez-Trujillo and colleagues in 2013 [9], and coincides with the SIV uptake rate of 53% issued by Zamorano-Leon et al. in 2020 [10]. The analysis with a multivariable logistic regression model showed that, against general expectations, higher education attainment, specifically graduate education, is associated with an increased likelihood of non-vaccination. These findings stand in contrast to a similar study performed in Hungary in 2023 [18], where tertiary education levels showed higher odds of being vaccinated, but align with a different study from South Korea in 2021 where higher educational attainment showed lower odds of being vaccinated [17], possibly being explained by a Halo effect or anchoring bias with regards to pwDMs with higher educational attainment and indicating a gap of attention towards these pwDMs. Social support also emerged as a significant factor, with varying levels of support influencing vaccination likelihood, suggesting a dose–response effect, highlighting the importance of community and family in health behaviours. To our knowledge, in the context of pwDMs, this was not shown before. Interestingly, the time since the last medical visit emerged as a significant predictor, with those visiting an HCP more than 4 weeks ago more likely to be unvaccinated, potentially and partially explained by a recall bias but underscoring the need for regular medical visits for pwDMs. Consultations with nurses or midwives and the use of cold medications were associated with higher vaccination rates, pointing to the influence of healthcare accessibility and health-seeking behaviour on vaccination decisions. These results align with the findings of a recent study in Israel from Dopelt et al. who found that trust in the healthcare system is a mayor predictor of vaccine uptake probability, underscoring the positive role that HCPs like nurses, midwives and pharmacists can play in recommending or administering the SIV and suggesting that these healthcare professionals are pivotal in enhancing vaccination coverage through their direct interactions with patients [26,27]. Also, economic barriers to medical care were found to negatively affect vaccination uptake. Efforts to mitigate economic barriers to healthcare could enhance vaccination rates, as previously described by Schmid et al. [28].

Recent flu vaccination campaigns have predominantly targeted the demographic aged 60 and above, reflecting a strategic emphasis on this cohort through the concerted development of audiovisual materials, including posters and infographics, across both national and regional health authorities [29,30,31]. Moreover, the standardised vaccination schedule, applicable across various age groups [24], predominantly references populations over the age of 65 or below 4, inadvertently marginalising the vaccination imperative for intermediary risk groups, notably pwDMs. This gap is ostensibly bridged in the seasonal flu and COVID-19 vaccination recommendations [32], which establish foundational guidelines subsequently adapted by autonomous communities in their respective health directives [30,33].

The age-specific prioritisation for vaccination, particularly among those aged 60 and above, is corroborated by findings from the ADVISE study [34] indicating a perceptual discrepancy among healthcare professionals regarding the uniform importance of vaccination across different age groups. This underscores an exigent need for enhanced vaccination-related training for healthcare providers, with a notable emphasis on addressing the prevalent scepticism and negative perceptions towards vaccination efficacy within the medical and nursing fraternities [35]. Such educational interventions are necessary for ameliorating the vaccination uptake rates within the country, particularly given that a significant proportion of vaccinations among patients with diabetes occur consequent to medical or nursing recommendations [36], highlighting the pivotal role of healthcare professionals in patient vaccination decisions.

These findings have important implications for public health policy and intervention strategies. Tailored educational programs targeting individuals with higher education levels and strengthening community support systems and enhancing accessibility to HCPs, including nurse-driven vaccination, as well as employing digital reminders through Electronic Health Records (EHRs), may also prove beneficial [37,38,39].

The limitations of our study include the reliance on self-reported data and the cross-sectional design, which limits our ability to establish causality. Although efforts have been made to maintain representativeness, analysing a subsample may introduce limitations regarding the national representativeness of the results. Similarly, the stratified analysis may have affected the power on some variables of interest even though several of them were grouped together to account for this issue. Self-reported data may introduce potential recall and social desirability bias, and there is a risk of underestimating DM rates. Previous research by Jiménez-García in 2014 suggests that self-reported vaccination rates tend to overestimate actual rates, possibly due to social desirability bias [14]. Therefore, it is possible that our reported vaccination rates are higher than reality. Additionally, this study does not differentiate between different subtypes of DM. Different types of self-indicated DM diagnoses could not be addressed due to the lack of statistical power. Also, this study did not include non-institutionalised individuals or those under 15 years of age. Further, parts of the EHIS 2020 were conducted during the first year of the COVID-19 pandemic, potentially leading to biases in data acquisition among participants with an unknown direction. Participants might have been influenced by reiterated public Spanish government influenza vaccination recommendations for chronically ill people, possibly leading to the over-reporting of influenza vaccination.

On the other hand, the strengths of our study lie in its utilisation of the most recent and nationally representative data available for Spain. Our large sample size enhances generalisability, and the robust sampling methodology minimises missing data. These strengths contribute to the overall reliability and validity of our findings.

## 5. Conclusions

Overall, this study contributes valuable insights into the factors affecting influenza vaccination among those with diabetes in Spain, offering a solid foundation for future targeted public health strategies. Based on our insights and to improve SIV rates among pwDMs, we recommend that Spanish Public Health Authorities implement tailored interventions such as targeted awareness campaigns specifically aimed at younger, more educated pwDMs, infrequent healthcare users and those facing economic barriers and low social support. Further research should be conducted to elucidate reasons for vaccine hesitancy among these identified groups. Educational programs should address the unique concerns and perceptions of highly educated individuals with DM. Efforts should be made to ensure that individuals with DM engage in regular healthcare interactions, emphasising the importance of annual medical visits. Mitigating economic barriers to healthcare access is essential to improve vaccination uptake, and strengthening community support systems while highlighting the role of family and social networks in health behaviours can significantly impact vaccination rates.

It is certainly beneficial to educate and engage HCPs about the necessity and benefits of SIV for pwDMs of all ages. The utilisation of EHRs and SMS for annual digital reminders for pwDMs and HCPs is also recommended. Additionally, it is important to investigate the reasons for non-adherence to vaccination recommendations among HCPs and pwDMs. Finally, efforts should be made to fill knowledge gaps concerning institutionalised pwDMs and those younger than 15 years of age.

## Figures and Tables

**Table 1:** Characteristics of survey respondents overall and according to immunisation status and age group. Only variables with *p* **≤** 0.2 are displayed in the table.

		Under 60 Years				Over 60 Years			
Variable	Categories	Overall N (%)	Vaccinated N (%) [CI 95%]	Unvaccinated N (%) [CI 95%]	*p*-Value	Overall N (%)	Vaccinated N (%) [CI 95%]	Unvaccinated N (%) [CI 95%]	*p*-Value
Sex					0.2				0.3
	Men	238 (100)	59 (25) [20–31]	179 (75) [69–80]		871 (100)	505 (58) [55–61]	366 (42) [39–45]	
	Women	188 (100)	58 (31) [24–38]	130 (69) [62–76]		896 (100)	541 (60) [57–64]	355 (40) [36–43]	
Spanish nationality/citizenship					0.4				0.13
	Yes	380 (100)	107 (28) [24–33]	273 (72) [67–76]		1752 (100)	1040 (59) [57–62]	712 (41) [38–43]	
	No	46 (100)	10 (22) [11–37]	36 (78) [63–89]		15 (100)	6 (40) [17–67]	9 (60) [33–83]	
Marital status					0.2				0.011
	Single	108 (100)	23 (21) [14–30]	85 (79) [70–86]		141 (100)	73 (52) [43–60]	68 (48) [40–57]	
	Married	261 (100)	77 (30) [24–35]	184 (70) [65–76]		924 (100)	550 (60) [56–63]	374 (40) [37–44]	
	Widowed	9 (100)	1 (11) [0.58–49]	8 (89) [51–99]		595 (100)	371 (62) [58–66]	224 (38) [34–42]	
	Divorced	48 (100)	16 (33) [21–49]	32 (67) [51–79]		106 (100)	51 (48) [38–58]	55 (52) [42–62]	
Study level					0.2				<0.001
	Incomplete Primary Education	33 (100)	14 (42) [26–61]	19 (58) [39–74]		508 (100)	317 (62) [58–67]	191 (38) [33–42]	
	Complete Primary Education	204 (100)	53 (26) [20–33]	151 (74) [67–80]		906 (100)	555 (61) [58–64]	351 (39) [36–42]	
	Graduate	87 (100)	26 (30) [21–41]	61 (70) [59–79]		172 (100)	82 (48) [40–55]	90 (52) [45–60]	
	Postgraduate	102 (100)	24 (24) [16–33]	78 (76) [67–84]		181 (100)	92 (51) [43–58]	89 (49) [42–57]	
Health perception					0.013				0.10
	Good	352 (100)	88 (25) [21–30]	264 (75) [70–79]		1362 (100)	792 (58) [55–61]	570 (42) [39–45]	
	Poor	74 (100)	29 (39) [28–51]	45 (61) [49–72]		405 (100)	254 (63) [58–67]	151 (37) [33–42]	
Health insurance					0.3				0.10
	Public	415 (100)	116 (28) [24–33]	299 (72) [67–76]		1715 (100)	1021 (60) [57–62]	694 (40) [38–43]	
	Private	11 (100)	1 (9.1) [0.48–43]	10 (91) [57–100]		52 (100)	25 (48) [34–62]	27 (52) [38–66]	
Time of last medical visit					<0.001				<0.001
	In the last 4 weeks	165 (100)	65 (39) [32–47]	100 (61) [53–68]		691 (100)	449 (65) [61–69]	242 (35) [31–39]	
	More than 4 weeks	261 (100)	52 (20) [15–25]	209 (80) [75–85]		1075 (100)	597 (56) [53–59]	478 (44) [41–47]	
Depressive severity					0.038				0.6
	None	323 (100)	78 (24) [20–29]	245 (76) [71–80]		1264 (100)	745 (59) [56–62]	519 (41) [38–44]	
	Moderate	81 (100)	31 (38) [28–50]	50 (62) [50–72]		387 (100)	232 (60) [55–65]	155 (40) [35–45]	
	Severe	9 (100)	2 (22) [3.9–60]	7 (78) [40–96]		24 (100)	12 (50) [31–69]	12 (50) [31–69]	
Social class					0.2				0.14
	Directors and managers	51 (100)	9 (18) [8.9–31]	42 (82) [69–91]		203 (100)	106 (52) [45–59]	97 (48) [41–55]	
	Intermediate and self-employed occupation workers	68 (100)	19 (28) [18–40]	49 (72) [60–82]		285 (100)	159 (56) [50–62]	126 (44) [38–50]	
	Skilled and semi-skilled workers in the primary sector	156 (100)	38 (24) [18–32]	118 (76) [68–82]		604 (100)	366 (61) [57–64]	238 (39) [36–43]	
	Supervisors and technical skilled workers	61 (100)	21 (34) [23–48]	40 (66) [52–77]		279 (100)	173 (62) [56–68]	106 (38) [32–44]	
	Unskilled workers	82 (100)	28 (34) [24–46]	54 (66) [54–76]		277 (100)	167 (60) [54–66]	110 (40) [34–46]	
Social support					0.2				0.013
	A lot	356 (100)	103 (29) [24–34]	253 (71) [66–76]		1540 (100)	930 (60) [58–63]	610 (40) [37–42]	
	Somewhat	43 (100)	10 (23) [12–39]	33 (77) [61–88]		163 (100)	83 (51) [43–59]	80 (49) [41–57]	
	Little or nothing	24 (100)	3 (13) [3.3–33]	21 (88) [67–97]		55 (100)	26 (47) [34–61]	29 (53) [39–66]	
Alcohol					0.009				0.019
	Never or not in the last 12 months	192 (100)	67 (35) [28–42]	125 (65) [58–72]		910 (100)	566 (62) [59–65]	344 (38) [35–41]	
	Once per month or less	70 (100)	15 (21) [13–33]	55 (79) [67–87]		234 (100)	138 (59) [52–65]	96 (41) [35–48]	
	More than once per week	162 (100)	35 (22) [16–29]	127 (78) [71–84]		622 (100)	342 (55) [51–59]	280 (45) [41–49]	
Tobacco					>0.9				0.014
	Yes	262 (100)	72 (27) [22–33]	190 (73) [67–78]		781 (100)	437 (56) [52–59]	344 (44) [41–48]	
	No	164 (100)	45 (27) [21–35]	119 (73) [65–79]		986 (100)	609 (62) [59–65]	377 (38) [35–41]	
No medical attention for economic barriers					0.8				0.007
	No	406 (100)	111 (27) [23–32]	295 (73) [68–77]		1732 (100)	1033 (60) [57–62]	699 (40) [38–43]	
	Yes	20 (100)	6 (30) [13–54]	14 (70) [46–87]		35 (100)	13 (37) [22–55]	22 (63) [45–78]	
Nurse or midwife consultation					0.2				<0.001
	Yes	108 (100)	35 (32) [24–42]	73 (68) [58–76]		576 (100)	378 (66) [62–69]	198 (34) [31–38]	
	No	318 (100)	82 (26) [21–31]	236 (74) [69–79]		1191 (100)	668 (56) [53–59]	523 (44) [41–47]	
Cold medications					0.13				0.045
	Yes	53 (100)	19 (36) [23–50]	34 (64) [50–77]		184 (100)	122 (66) [59–73]	62 (34) [27–41]	
	No	355 (100)	92 (26) [22–31]	263 (74) [69–78]		1533 (100)	899 (59) [56–61]	634 (41) [39–44]	
Use of emergency services					0.9				0.14
	Yes	129 (100)	36 (28) [21–37]	93 (72) [63–79]		502 (100)	311 (62) [58–66]	191 (38) [34–42]	
	No	297 (100)	81 (27) [22–33]	216 (73) [67–78]		1265 (100)	735 (58) [55–61]	530 (42) [39–45]	
Hospitalisation					0.5				0.2
	Yes	51 (100)	16 (31) [20–46]	35 (69) [54–80]		305 (100)	190 (62) [57–68]	115 (38) [32–43]	0.3
	No	375 (100)	101 (27) [23–32]	274 (73) [68–77]		1462 (100)	856 (59) [56–61]	606 (41) [39–44]	

**Table 2:** Adjusted multivariable logistic regression analysis of factors influencing influenza vaccination uptake. Only variables significant in the multivariable stepwise logistic regression are displayed in this table.

	Under 60 Years			Over 60 Years		
Characteristic	OR *	95% CI	*p*-Value	OR	95% CI	*p*-Value
Sex						
Men	Ref.					
Women	0.74	0.46, 1.18	0.2	1.02	0.83, 1.25	0.9
Study level						
Incomplete primary education	Ref.					
Complete primary education	3.53	1.49, 8.38	0.004	0.96	0.76, 1.22	0.8
Graduate	3.33	1.31, 8.61	0.012	1.78	1.24, 2.57	0.002
Postgraduate	3.77	1.45, 9.38	0.005	1.40	0.98, 2.02	0.066
Time of last medical visit						
In the last 4 weeks	Ref.					
More than 4 weeks	1.03	0.44, 2.40	>0.9	2.31	1.59, 3.38	<0.001
No medical attention for economic barriers						
No	Ref.					
Yes	1.19	0.42, 3.76	0.8	2.66	1.32, 5.56	0.007
Social support						
A lot	Ref.					
Somewhat	1.44	0.63, 3.75	0.4	1.48	1.06, 2.07	0.023
Little or nothing	2.91	0.85, 13.3	0.13	1.82	1.06, 3.18	0.034
Nurse or midwife consultation						
Yes	Ref.					
No	0.77	0.36, 1.61	0.5	2.22	1.56, 3.19	<0.001
Cold medications						
Yes	Ref.					
No	1.69	0.86, 3.26	0.12	1.33	0.95, 1.88	0.1

* Adjusted Odds ratio (OR) from logistic regression.

## Data Availability

The data presented in this study are available on the website from the Spanish national statistics institute (Instituto Nacional de Estatística—INE): https://www.ine.es/dyngs/INEbase/es/operacion.htm?c=Estadistica_C&cid=1254736176784&menu=resultados&idp=1254735573175#tabs-1254736195298 (last seen on 28 May 2024).

## Supplementary Materials

The following supporting information can be downloaded at https://www.mdpi.com/article/10.3390/vaccines12080915/s1, Table S1: Unadjusted Logistic Regression Analysis of Factors Influencing Influenza Vaccination Uptake; Table S2: Collinearity test in final model for strata less than 60 years old; Table S3: Collinearity test in final model for strata over 60 years old; Table S4: Immunisation status in different age groups in study population; Table S5: Immunisation status on different diabetes-related variables in the study population; Figure S1: Study design flowchart.
