# Supplementary material for: Influenza Vaccination Uptake and Associated Factors among Individuals with Diabetes Mellitus in Spain: A Cross-Sectional Study Using Data from the European Health Interview Survey 2020
Source: Vaccines (Basel). 2024 Aug 13;12(8):915. doi: 10.3390/vaccines12080915 (PMC11359187; doi:10.3390/vaccines12080915)
Supplement: Supplementary file 1 [file vaccines-12-00915-s001.zip › vaccines-3119616-supplementary.pdf]

Supplementary Table S1. Unadjusted Logistic Regression Analysis of Factors Influencing Influenza Vaccination Uptake.

	Under 60 years			Over 60 years		
**Characteristic**	**OR**	**95% CI**	**p-value**	**OR**	**95% CI**	**p-value**
Sex						
Men						
Women	0.67	0.43, 1.04	0.071	0.90	0.74, 1.10	0.3
Study level						
Incomplete Primary Education						
Complete Primary Education	2.21	1.01, 4.76	0.043	1.03	0.82, 1.29	0.8
Graduate	1.94	0.83, 4.53	0.12	1.84	1.29, 2.63	<0.001
Postgraduate	2.54	1.09, 5.90	0.030	1.52	1.07, 2.16	0.019
Time of last medical visit						
In the last 4 weeks						
More than 4 weeks	2.81	1.80, 4.41	<0.001	1.49	1.22, 1.83	<0.001
No medical attention economical barriers						
No						
Yes	0.87	0.34, 2.52	0.8	2.41	1.21, 4.96	0.014
Social support						
A lot						
Somewhat	1.65	0.77, 3.95	0.2	1.43	1.03, 1.98	0.032
Little or nothing	2.74	0.91, 11.8	0.11	1.72	1.00, 2.97	0.049
Nurse or midwife consultation						
Yes						
No	1.48	0.91, 2.39	0.11	1.49	1.21, 1.85	<0.001
Cold medications						
Yes						
No	1.64	0.88, 3.00	0.11	1.40	1.02, 1.95	0.040

Supplementary Table S2. Collinearity test in final model for strata less than 60 years old.

**Covariate**	**GVIF**
Sex	1.03
Study level	1.13
Time of last medical visit	3.39
No medical due to attention economical barriers	1.03
Social support	1.08
Nurse or midwife consultation	2.21
Cold medications	1.03
Interaction term (Time of last medical visit:Nurse or midwife consultation)	4.28

Supplementary Table S3. Collinearity test in final model for strata over 60 years old.

**Covariate**	**GVIF**
Sex	1.05
Study level	1.07

Time of last medical visit	3.38
No medical attention economical barriers	1.00
Social support	1.02
Nurse or midwife consultation	2.79
Cold medications	1.02
Interaction term (Time of last medical visit:Nurse or midwife consultation)	5.05

Supplementary Table S4. Immunisation status in different age groups in study population.

Covariable	Overall, N = 2,193<br>N (%)	Vaccinated, N = 1,163<br>% (IC 95)	Unvaccinated, N = 1,030<br>% (IC 95)	P - value
Age group				<0.001
15 - 19	6 (100)	17 (0.88 - 64)	83 (36 – 99)	
20 – 39	49 (100)	20 (11 – 35)	80 (65 - 89)	
40 – 59	371 (100)	29 (24 - 34)	71 (66 - 76)	
> 60	1,767 (100)	59 (57 – 61)	41 (39 - 43)	

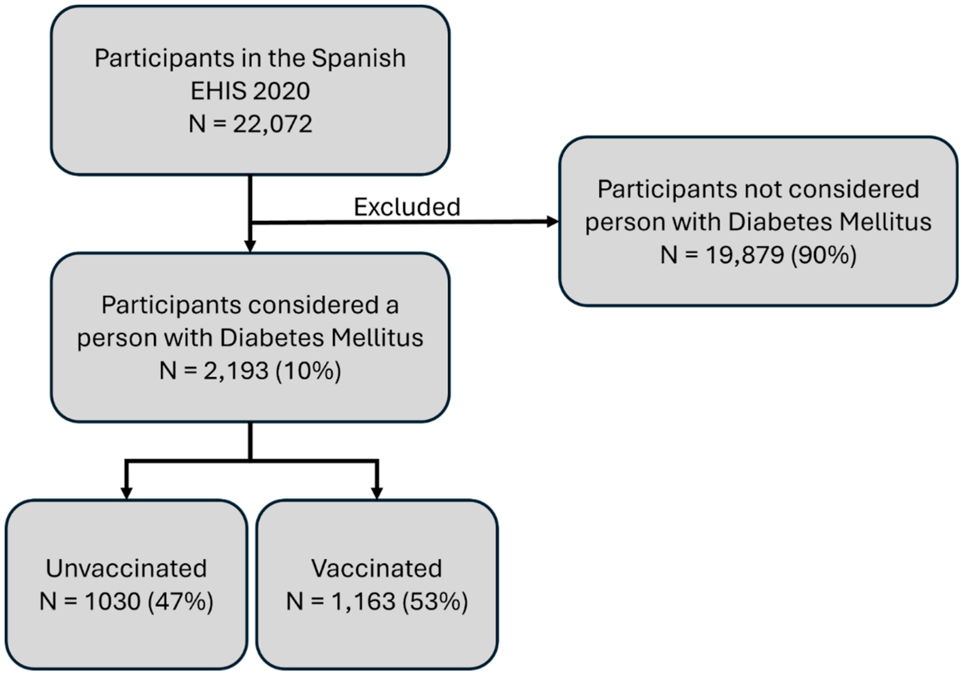

Figure S1: Study design flowchart.

The final sample size for the study was 2,193 individuals with diabetes, with a significance level (alpha) of 0.05 and a resulting statistical power of 0.80.

Supplementary Table S5. Immunisation status on different diabetes-related variables in the study population.

Covariable	Overall (n)	Vaccinated (n)	Unvaccinated (n)
Medication for diabetes	1937	1053	884
Having had a former diagnosis of DM	2166	1146	1020
Having a diagnosis of DM	2090	1111	979
All variables	2193	1163	1030
